# P-748. Evaluation of Risk Factors for Short Inpatient Hospital Stays for the Treatment of Cellulitis

**DOI:** 10.1093/ofid/ofae631.944

**Published:** 2025-01-29

**Authors:** Cole Orlikowski, Kyle Schmidt, Andrew Jameson, Lisa E Dumkow

**Affiliations:** Ferris State University College of Pharmacy, Grand Rapids, Michigan; Ferris State University College of Pharmacy, Grand Rapids, Michigan; Trinity Health Grand Rapids, Grand Rapids, Michigan; Trinity Health Grand Rapids, Grand Rapids, Michigan

## Abstract

**Background:**

Cellulitis is a common cause of hospitalization and imposes significant financial burden on healthcare systems. This study aimed to identify patient risk factors associated with short-stay hospitalizations for cellulitis with a goal to develop a dalbavancin protocol for use in the emergency department (ED) to mitigate short hospital stays.

**Figure:**
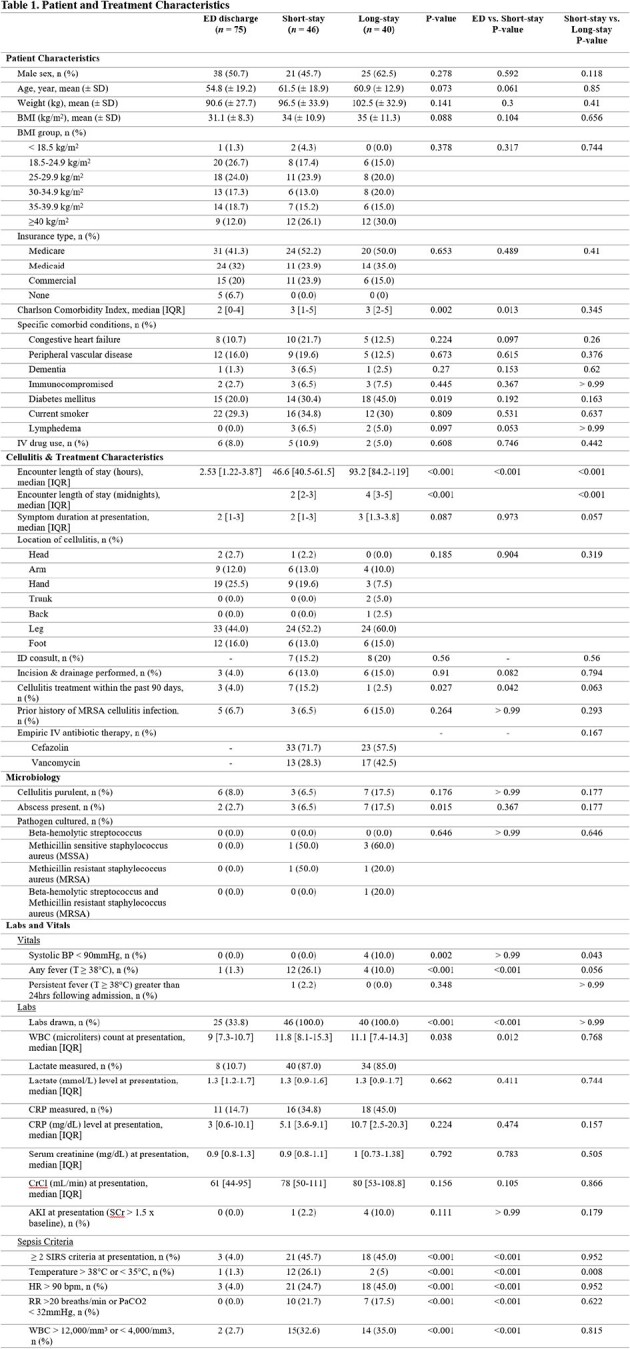

**Methods:**

A retrospective cohort study was conducted at a 350-bed community teaching hospital in Michigan evaluating adult patients diagnosed with cellulitis February 1, 2020 - August 1, 2023, who received initial treatment with monotherapy targeting beta-hemolytic streptococci or *Staphylococcus aureus*. Three cohorts were identified based on length of hospitalization or ED discharge. The primary outcome was to identify risk factors associated with short-stay (< 72 hour) hospitalizations. Multivariate logistic regression was used to identify independent patient factors associated with short-stay hospitalization in contrast to long-stay (≥ 72 hours) or ED discharge. Patients were excluded if they required surgical intervention, received polymicrobial therapy, required ICU admission, received cellulitis treatment in the past 90 days, or were being treated for multiple infections.

**Results:**

A total of 161 patients were included (ED discharge, n = 75; Short-stay, n = 46; and Long-stay, n = 40). Patient and treatment characteristics are summarized in Table 1. Logistic regression demonstrated that need for incision and drainage (I&D) (OR 7.23; 95% CI 1.69-38.04), Charlson Comorbidity Index ≥ 3 (OR 1.23; 95% CI 1.04-1.46), or presenting with fever (OR 31.62; 95% CI 5.67 – 596.30) were independent risk factors for short-stay hospitalization compared to ED discharge. Charlson Comorbidity Index > 3 (OR 1.25, 95% CI 1.06-1.48) and purulent cellulitis (OR 4.29, 95% CI 1.22-15.38) were predictive of long-stay hospitalizations. Characteristics such as fever, recurrent cellulitis, history of MRSA, and BMI were not associated with longer hospital stays.

**Conclusion:**

A protocol to evaluate ED patients with cellulitis for dalbavancin use could reduce unnecessary hospitalizations and healthcare cost. Patients with more comorbidities, purulent cellulitis needing I&D, and those with unstable vital signs may be less ideal candidates for ED dalbavancin use.

**Disclosures:**

**All Authors**: No reported disclosures

